# Automatic classification of tissues on pelvic MRI based on relaxation times and support vector machine

**DOI:** 10.1371/journal.pone.0211944

**Published:** 2019-02-22

**Authors:** Jorge Arturo Zavala Bojorquez, Pierre-Marc Jodoin, Stéphanie Bricq, Paul Michael Walker, François Brunotte, Alain Lalande

**Affiliations:** 1 Le2i, Université Bourgogne Franche-Comte, Dijon, France; 2 VITAL, Université de Sherbrooke, Sherbrooke, Canada; 3 Centre Hospitalier Universitaire, Dijon, France; Charite Universitatsmedizin Berlin, GERMANY

## Abstract

Tissue segmentation and classification in MRI is a challenging task due to a lack of signal intensity standardization. MRI signal is dependent on the acquisition protocol, the coil profile, the scanner type, etc. While we can compute quantitative physical tissue properties independent of the hardware and the sequence parameters, it is still difficult to leverage these physical properties to segment and classify pelvic tissues. The proposed method integrates quantitative MRI values (T1 and T2 relaxation times and pure synthetic weighted images) and machine learning (Support Vector Machine (SVM)) to segment and classify tissues in the pelvic region, i.e.: fat, muscle, prostate, bone marrow, bladder, and air. Twenty-two men with a mean age of 30±14 years were included in this prospective study. The images were acquired with a 3 Tesla MRI scanner. An inversion recovery-prepared turbo spin echo sequence was used to obtain T1-weighted images at different inversion times with a TR of 14000 ms. A 32-echo spin echo sequence was used to obtain the T2-weighted images at different echo times with a TR of 5000 ms. T1 and T2 relaxation times, synthetic T1- and T2-weighted images and anatomical probabilistic maps were calculated and used as input features of a SVM for segmenting and classifying tissues within the pelvic region. The mean SVM classification accuracy across subjects was calculated for the different tissues: prostate (94.2%), fat (96.9%), muscle (95.8%), bone marrow (91%) and bladder (82.1%) indicating an excellent classification performance. However, the segmentation and classification for air (within the rectum) may not always be successful (mean SVM accuracy 47.5%) due to the lack of air data in the training and testing sets. Our findings suggest that SVM can reliably segment and classify tissues in the pelvic region.

## Introduction

Tissue segmentation and classification is an important topic in Magnetic Resonance Imaging (MRI). It helps to study anatomical structures, to develop surgical planning, plan radiation therapy and perform quantitative analyses [1,2].

Most of the available segmentation and classification methods operate on conventional contrast-weighted MR images (T_1_-, T_2_-, and proton density-weighted images). However, the segmentation and classification of tissues from contrast-weighted MR images is fundamentally complex. This is due to the specific sequences used which impact the grayscale values of the resulting MR images. So, as opposed to CT-scan images, one cannot know *a priori* the grayscale values of a given tissue without acute knowledge of the exact acquisition protocol. As such, MR images are highly sensitive to acquisition parameters [3] such as the inversion time (TI), the echo time (TE), the repetition time (TR), the flip angle and the voxel size, as well as the sequences used to acquire the images [4]. MR images are also affected by various signal artifacts like partial volume effects, transverse coherences or spoiling, B_0_- and B_1_-inhomogeneities [5], as well as hardware characteristics (e.g. intra- and inter-scanner variations, magnetic field intensity, coil sensitivity [6]). Furthermore, the tissue properties themselves have an impact on the acquired MR images (e.g. relaxation times, proton density and physiological parameters). The interaction of these factors determines the image voxel intensity and, therefore, makes automatic tissue segmentation and classification challenging, especially for unsupervised methods [7].

Furthermore, the complexity of image formation leads to an unfortunate loss of physical meaning of the absolute voxel intensity [8]. This is because MR images are acquired in arbitrary units that are not comparable between studies [9]. Hence, segmentation and classification are performed by comparing contrast differences among arbitrary voxels’ intensities. As the intensity scaling is also arbitrary, MRI segmentation and classification methods require the implementation of complicated filtering and normalization techniques [10,11], which add additional steps to the segmentation and classification process. Moreover, these methods often have a strong heuristic flavor and do not generalize well to images that violate the hypotheses they are built upon.

To avoid these difficulties, some automatic methods work directly on parametric maps (quantitative MRI) or on generated synthetic images based on intrinsic properties of tissues such as T_1_ and T_2_ relaxation times. This is possible because in the T_1_ and T_2_ parametric maps, the voxel intensities represent individual tissue physical properties. These properties are scanner and pulse sequence independent, and have numerical meaning rather than representing signal intensity on an arbitrary scale [3,12]. i.e. quantitative MRI has the benefit of being independent of MRI settings and hardware imperfections [8]. A further advantage of the integration of imaging physics into the classification process is that it allows the optimization of the MR pulse parameters in a way that reduces the probability of misclassification [3].

Various segmentation and classification methods using the tissues’ physical properties have been proposed. For example, Chen et al. [13] performed segmentation of white matter (WM), gray matter (GM), and cerebrospinal fluid (CSF) using a region-based active contour method applied on T_1_ relaxation maps. Traynor et al. [14] utilized both T_1_ and T_2_ relaxation times to segment the thalamus in 16 regions using a genetic algorithm. Iglesias et al. [15] implemented an atlas-based method using generated synthetic images from the T_1_ relaxation times to segment the hippocampus. Knowing that the intensities of T_1_-weighted, T_2_-weighted or proton density images are the product of different physical variables, Iglesias et al. [15] computed synthetic T_1_-weighted images using the tissues’ physical properties to achieve image homogenization and perform atlas matching to segment the hippocampus. As mentioned by the authors, the drawback of their method is that the T_1_ value of at least one tissue must be known a priori. This is a major weakness considering that the true relaxation times of tissues are still not measured with sufficient accuracy and precision [5].

To the best of our knowledge, most previous methods have been designed for brain and thorax imaging, and little effort has been devoted to pelvic imaging, although prostate cancer is the most commonly diagnosed cancer for men worldwide [16]. For prostate cancer, MRI is currently the imaging modality of choice for early detection and classification of tumorous areas. An accurate segmentation and classification of prostate cancer is required to guide radiotherapy or surgery, to perform volume estimation and to track disease progression [17]. However, the segmentation and classification of prostate is particularly challenging in MRI because signal intensity is not standardized, and image appearance is for a large part determined by acquisition protocol, coil profile, scanner type and field strength [18]. These are major obstacles in the development of prostate segmentation and classification methods.

In this paper, we use a machine learning method to learn the relation between the T_1_/ T_2_ intrinsic parameters for six different tissues namely prostate, fat, muscle, bladder, bone marrow, and air (within the rectum). As such, a Support Vector Machine (SVM) is used to segment and classify the relevant structures within the pelvis. The MRI feature descriptors are the T_1_ relaxation time, the synthetic generated pure T_1_-weighted images (with different TI), the T_2_ relaxation time and synthetic generated pure T_2_-weighted images (with different TE), and a probabilistic shape prior which encodes the location of anatomical regions.

## Materials and methods

**Fig 1:**
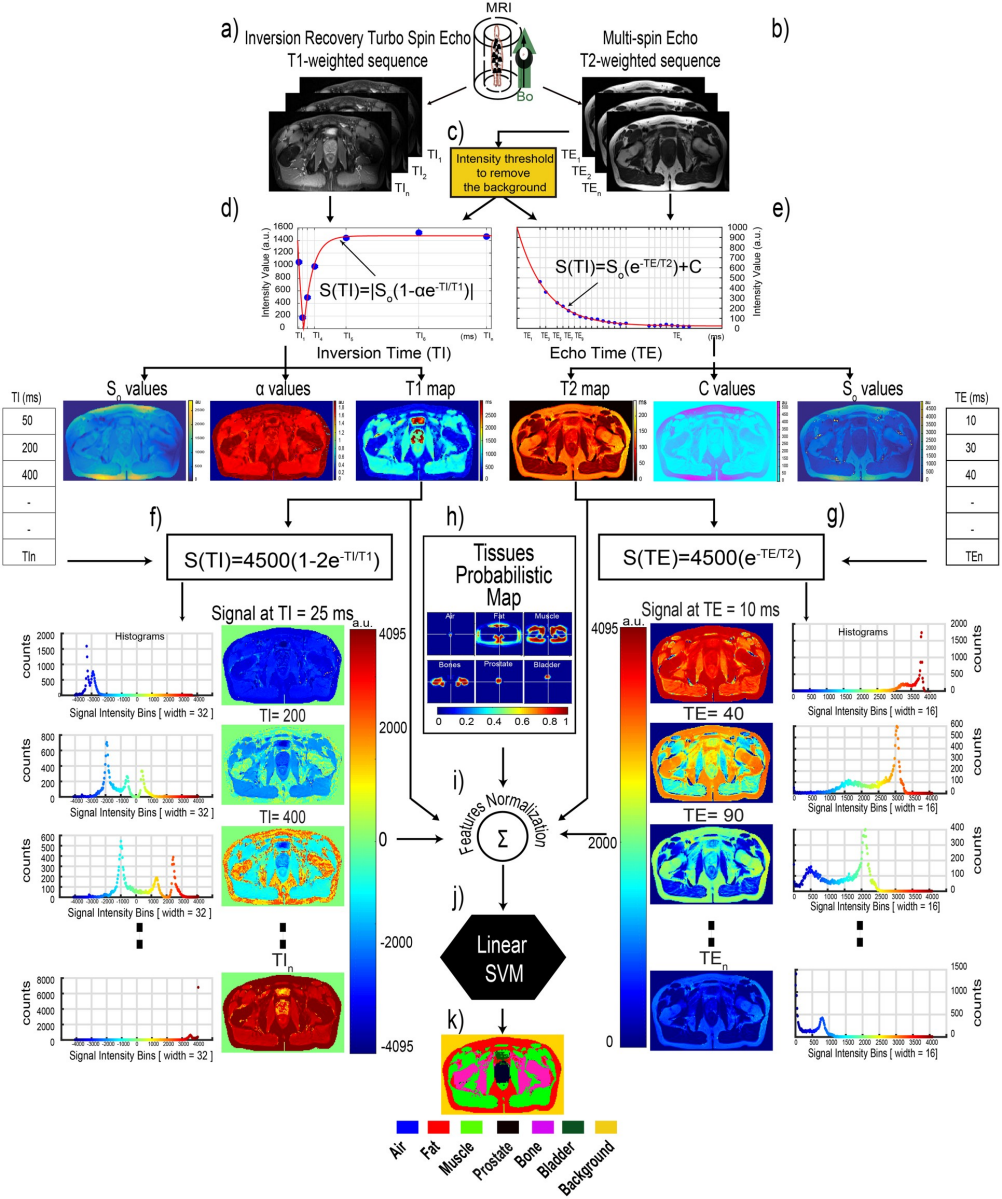
Pipeline of the segmentation and classification process using the SVM classifier. After obtaining the T_1_- and T_2_-weighted images (a-b) from their corresponding sequences, the body region is found using a threshold method on the T_2_-weighted images (c). Then, the fit procedure is applied to obtain the relaxation maps and other different variables from the received signal model (d-e). These variables are then used to generate the synthetic T_1_- and synthetic T_2_-weighted images (f-g). Later, an independent process using the training data is used to generate tissues probabilistic maps, i.e. the probability of a voxel belonging to different tissues (h). At this point, all feature descriptors are normalized (i) to be used by the multi-class SVM classifier (j), which predicts the different classes (k).

To carry out the segmentation and classification of the different tissues present in an image, our approach exploits the information obtained from different standard MR sequences. The sequences are used to obtain T_1_-weighted and T_2_-weighted images in order to compute the T_1_ and T_2_ relaxation maps. These maps serve as the input feature vectors of the SVM used for tissue segmentation-classification. Furthermore, the relaxation times are utilized to generate pure synthetic T_1_- and T_2_-weighted images that are also used as input features. A shape probabilistic map is also used as a feature descriptor to include anatomical information in the segmentation-classification method. The segmented image contains the labels and boundaries of the various tissues within. The different steps of our method are illustrated in Fig 1 on one slice of a volunteer.

### Image acquisition

The images were acquired with a 3T Trio TIM clinical scanner (Siemens Medical Solutions, Germany) using a phased-array body coil. Twenty-two men gave their informed consent to participate in the study, which had been approved by the local institutional review board and the local ethic committee (the IRB of the University Hospital of Dijon (France) approved the research). The French law and regulation was followed, and the management of the patient is endorsed by the University Hospital of Dijon, that assume having participant consent by default when the participant is supervised by our institution. The study procedures were in accordance with the ethical standards of the committees with responsibility for human experimentation and with the Helsinki Declaration of 1975, as revised in 2008. Certain pathologies may alter tissue relaxation values and so subjects with systemic pathologies such as hematological diseases (anemias, myelodysplasias) and muscular disorders (atrophy, Duchenne and Becker diseases, etc.) should be excluded from the study. The group was comprised of 22 healthy volunteers. The age range was from 22 to 63 years old (mean 30 ± 14). Four to five slices were acquired depending on the anatomy of the subject. MRI slices were acquired with the same FOV, resolution and position at the level of the pelvis for both T_1_- and T_2_-weighted images. Axial pelvic orientations were chosen as it is commonly used in prostate MRI.

An inversion recovery-prepared turbo spin echo sequence was used to obtain the T_1_-weighted images. These images were acquired at eight different inversion times (i.e. TIs were from 50 ms to 10000 ms). The TIs were chosen to assure maximum contrast among tissues and to cover the whole span of T_1_ relaxation times of tissues present in the slice section. Other nominal sequence parameters are repetition time (TR) of 14000 ms, echo time (TE) of 7.4, echo train length (ETL) of 11, field of view (FOV) from 180 x 280 mm to 220 x 380 mm, slice thickness of 5 mm, number of excitations (NEX) = 1 and matrix size of 168 x 256. The average acquisition time per TI was 3 min 33 s.

A 32-echo spin echo sequence was used to obtain the T_2_-weighted images. The T_2_-weighted images were acquired at different TE from 8.8 to 281.6 ms in 8.8 ms steps with a TR of 5000 ms. The same nominal values for FOV, NEX and matrix size were defined as for the T_1_ sequence of every volunteer. The acquisition time was 14 minutes for the T_2_-weighted spin echo sequence.

### Segmentation and classification

In our method, MR images are segmented by classifying each pixel with a multi-class linear SVM classifier [19]. As mentioned in the introduction, the grayscale values of a given tissue can vary significantly from one acquisition to another. As such, raw input data suffer from a large variance making it hardly separable. While non-linear classifiers such as random forests [20], Adaboost [21], neural networks [22] or Kernel-SVM [23] could be used, the large variance of the data can lead to overfitting and poor generalization, especially when the training set accounts for a limited number of subjects. In this paper, we use features with a much lower variance while making classes linearly separable. While linear models are less prone to overfitting (and thus ensure a lower generalization error) they are also much faster, easier to train and have fewer hyper-parameters. The features used are the T_1_ relaxation time, the synthetic T_1_-weighted images, the T_2_ relaxation time, the synthetic T_2_-weighted images, and a probabilistic shape prior, which are explained in the following section.

**Fig 2:**
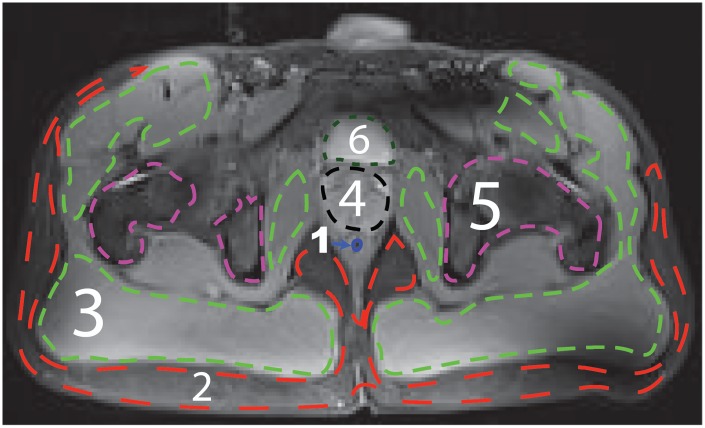
Selected ROIs on a T_1_-weighted image of the pelvic area. 1) air, 2) fat, 3) muscle, 4) prostate, 5) bone marrow, and 6) bladder.

The labels of the training data were defined by manually selecting ROIs corresponding to the different tissues present in the imaged anatomical areas, i.e. prostate, fat, muscle, bone marrow, bladder and air. ROIs were outlined by a radiologist with 20 years of experience based on a T_1_- and/or T_2_-weighted image that best depicted the tissues. Selected ROIs and their corresponding labels are illustrated in Fig 2.

### T_1_ relaxation time

The longitudinal relaxation time, T_1_ feature, is calculated from a magnitude monoexponential fit to the signal recovery data for each voxel using the bisquare weights nonlinear least squares fitting method [24]. This method was chosen for its capacity to eliminate the influence of noise by reducing the weight of outliers during the fit. A three-parameter model was used to describe the received signal at different inversion times (S(TI)):

$$S(TI)=|S_{0}(1-{\alpha e}^{-TI/T_{1}})|$$ (1)

where T_1_ is the relaxation time, S_0_ the equilibrium magnetization and α the inversion efficiency. These parameters represent the fitting variables of the model. S(TI) is the measured signal value for a given value of TI. The model describes a magnitude signal (only positive values) because the phase component of the received signal is not used during the reconstruction (i.e. the acquired MR images were magnitude images with no phase component).

The fitting interval restrictions on the model were set to the values defined in [24] (S_0_ [1, 65535], α[0, 2] and T_1_[0, 3000]). The interval on S_0_ considers any potential signal intensity expected in a MR DICOM image, the α intervals span all the potential inaccuracies of the scanner inversion pulse from 0° to 180°, and the T_1_ intervals span all the expected T_1_ relaxation times of tissues present in the image.

To reduce the fitting time, the background is automatically extracted and the fit is performed only with voxels that belong to the body region. This is achieved by creating a body mask from the T_2_-weighted image with the shortest TE (8.8 ms) with a threshold of 4% of the highest voxel intensity. At this TE, the tissues have almost full transversal magnetization and their intensity is maximum (S_tissue_≈25*S_background or noise_), which allows an easy separation between the body region and the background. The body region is defined by performing connected component analysis, i.e. two regions are identified and the region with the largest number of voxels is labeled as the body region (Size_body region_≈ 4 Size_background_).

The accuracy of the fit was measured by the R_square_ metric. Only the R_square_ values of voxels describing ≥85% of the data were considered as good fits. The fitting stops when a maximum number of iterations is reached or a good fit is found. Fig 1D shows the process of the T_1_ relaxation time calculation.

Since the fit was performed in a voxel by voxel fashion, the obtained relaxation times formed a T_1_ relaxation map which has the appearance of an image (structures can be identified), conceptually different from a T_1_-weighted image, in that individual pixel values now have a numerical meaning (i.e. T_1_ values in ms at each location in the anatomical area), rather than representing signal intensity on an arbitrary scale.

Once the parameters from the fit are calculated, the inverse process is performed to obtain a synthetic T_1_-weighted image.

### Synthetic T_1_-weighted images

Synthetic MR images can be generated with arbitrary contrast weighting, if the appropriate MR signal model is used [25]. In this case, the synthetic T_1_-weighted images are generated using the following model:

$$S(TI)=S_{0}(1-{2e}^{-TI/T_{1}})$$ (2)

**Fig 3:**
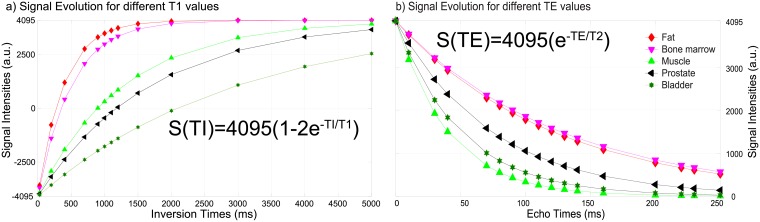
Model of a T_1_ and T_2_ relaxation signal. The T_1_ relaxation curve is simulated with an ideal inversion pulse (α = 2 = 180°) and magnetic field homogenization (S_0_ = 4095) (a). The T_2_ signal is also simulated with magnetic homogenization (S_0_ = 4095). Reported T_1_ and T_2_ values at 3 Tesla from the literature were used to generate the signal: fat (385 ms, 121ms), bone marrow (585 ms, 127 ms), muscle (1295 ms, 40 ms), prostate (1700 ms, 74 ms), and bladder (3000 ms, 50 ms) [24,33,34], respectively. TIs and TEs were chosen to cover the complete span of relaxation times of the previous tissues.

This model describes a signal with a perfect inversion pulse of 180° (i.e. α = 2) and depends on only three variables: S_0_, T_1_ and TI. An example of the signal modeling is shown in Fig 3A.

**Fig 4:**
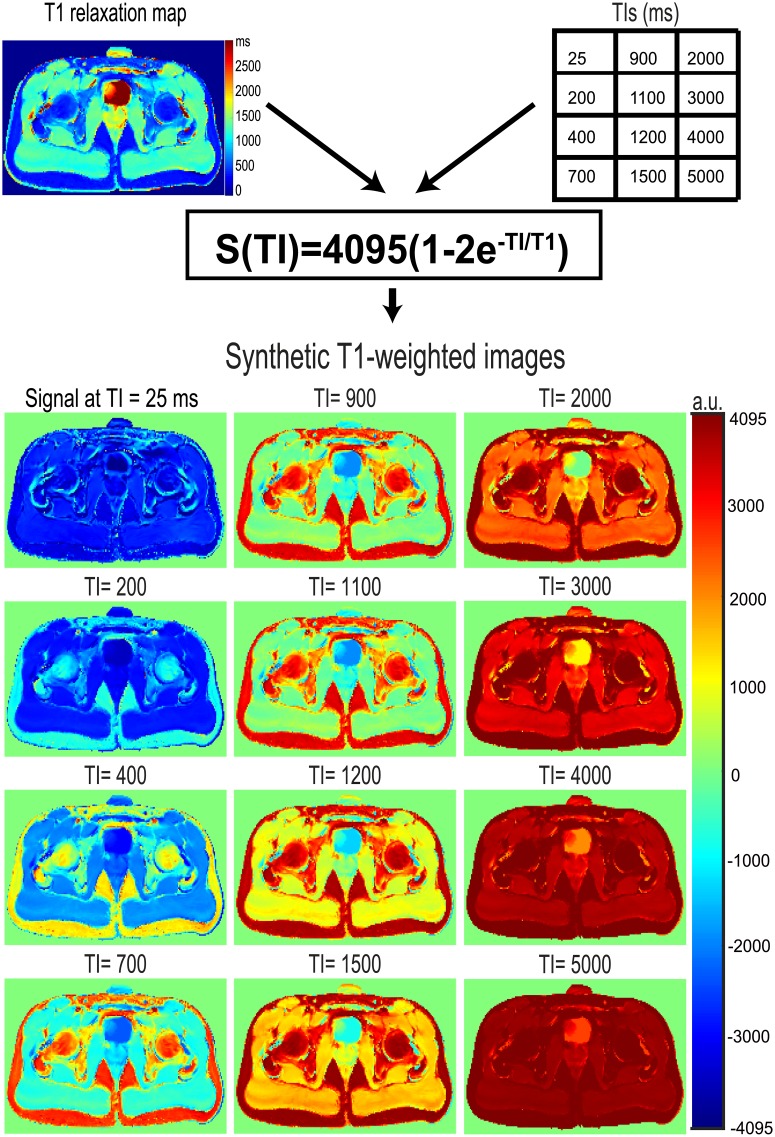
Process by which the synthetic T_1_-weighted image is computed. The relaxation times of tissues and the selected inversion times (TI) are used to generate the synthetic T_1_-weighted images using the model describing a received signal from a perfect inversion pulse.

To generate the synthetic T_1_-weighted images, S_0_ is assigned a constant maximum intensity value commonly used by MR scanners [26] (i.e. 4095). This constant value simulates a perfect homogeneous B_0_ for every voxel. The T_1_ relaxation values are set from the values found from the T_1_ fit calculation. The TIs were defined to span all the relaxation times of tissues present in the image, and to sample the signal intensities where they could produce the maximum contrast among tissues (TIs = 25 ms, 200 ms, 400 ms, 700 ms, 900 ms, 1000 ms, 1100 ms, 1200 ms, 1500 ms, 2000 ms, 3000 ms, 4000 ms, 5000 ms). Examples of generated synthetic T_1_-weighted images are provided in Fig 4.

The idea behind the chosen TIs is to sample the relaxation signal of tissues at precise moments where signal differences are maximal among tissues (e.g. at TI = 1200 ms, Fig 4), and to avoid sampling at those TIs where the signal of tissues are not easy to differentiate. However, it is difficult to define the perfect TIs a priori because the precise T_1_ relaxation times of tissues are still not measured with sufficient accuracy and precision [5]. A practical workaround is to use many TIs to cover a large span of T_1_ relaxation times (and by focusing on TI values that maximally differentiates tissues). This is because short TIs sample points favor the short T_1_ relaxation times, and long TIs sample times favor the long T_1_ relaxation times [8].

The synthetic T_1_-weighted images have important advantages with respect to the T_1_-weighted images: 1) The proton density influence and the effects of B_0_ and B_1_ inhomogeneities are removed; 2) The evolution of the signal has now a phase and not only a magnitude (i.e. it goes from negative to positive values). This is important because in magnitude images, tissues with different T_1_ relaxation times could be mapped with the same signal intensity (e.g. a signal intensity of 50 is equal to |-50| in magnitude images); 3) There is image homogenization through all the synthetic T_1_-weighted images (i.e. all intensities are in the range of [-4095, 4095]); 4) The dynamic range doubles with respect to the acquired T_1_-weighted images (i.e. from [0, 4095] to [-4095, 4095]); and 5) The synthetic images are independent of the scanner hardware and acquisition protocol.

### T_2_ relaxation time and synthetic T_2_-weighted images

For the T_2_ relaxation time, a mono-exponential fit using a bisquare weights nonlinear least squares fitting method was applied to the data [24]. The following three-parameter model was used to describe the received signal at different echo times (S(TE)):

$$S(TE)=S_{0}e^{-TE/T_{2}}+C$$ (3)

where T_2_, S_0_ and the y-offset (noise floor C) are the fitting parameters [27].

The fit was calculated only for voxels belonging to the region of the body defined during the T_1_ processing. The fits of voxels with R_square_ ≥ 85% were kept. The model interval restrictions were defined to reduce the fitting time (S_0_ [1, 65535], T_2_ [0, 3500] and C [0, 5000]). Fig 1E shows the process of the T_2_ relaxation time calculation.

Once the parameters from the fit are calculated, the inverse process is performed to obtain a synthetic T_2_-weighted image. This inverse process is similar to the one used to generate the synthetic T_1_-weighted image. The synthetic T_2_-weighted images are generated using the model described in Eq 3. S_0_ is kept constant (4095, a common maximum intensity value found in MR images), without y-offset (C = 0), and the T_2_ relaxation values are set from the values found from the T_2_ fit calculation. An example of the signal modeling is shown in Fig 3B.

As for the synthetic T_1_-weighted image, it is difficult to define the perfect TEs a priori, as many TEs were used to cover a large span of T_2_ relaxation times. This is because short TEs sample points favor the short T_2_ relaxation times, and long TEs sample points favor the long T_2_ relaxation times [8].

The synthetic T_2_-weighted images have important advantages with respect to the T_2_-weighted images: 1) The effects of B_0_ inhomogeneities, B_1_ inhomogeneities, proton density influence and noise (C) are removed; and 2) There is signal intensity homogenization (i.e. all the synthetic images are generated using the same signal scale: 0 to 4095).

### Shape prior

In order to enforce anatomically plausible results, we created a probabilistic shape prior *P*(*C*|*x*) which encodes the posterior probability of having a tissue of a certain class C given a voxel position x. The process for computing this shape prior consists in the following steps:

1. We localize the body center of mass for every volunteer in the training set.
2. Every voxel in the ROIs drawn by the expert is translated into an accumulator grid using the body center of mass as the reference point. Each bin in the accumulator grid records the number of tissue instances for the different tissues. The accumulator grid has a size of 80 x 80 cm^2^ and covers all possible variations. The bin dimensions of the grid are set to 5 x 5 mm^2^, a trade-off between the partial volume effects (bigger bins increase the possibility of this effect) and the computation time (smaller bins increase the computation time and the amount of memory to handle the probabilistic maps).
3. Finally, the probability that a certain voxel belongs to a particular tissue is computed for every bin by averaging out the ground truth labels of each training image.

**Fig 5:**
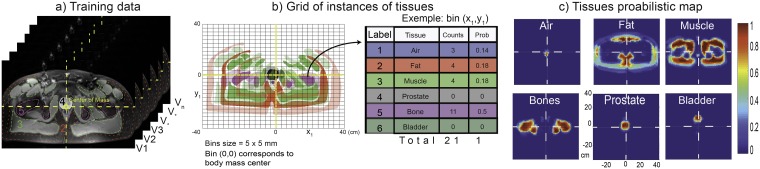
Probabilistic shape prior creation. Starting from training images, the center of mass is localized (a). Then, every voxel in the ROIs is translated into an accumulator grid (b) and the instances of every different tissue in the different bins are counted to create the probabilistic map (c). From the probabilistic map, six-features descriptors are formed: air, fat, muscle, bone marrow, prostate and bladder.

The process is illustrated in Fig 5. The posterior probability values from the shape prior will later on be used as feature descriptors.

### Feature normalization

At this point, we have 36 features: one T_1_ relaxation time, one T_2_ relaxation time, 12 synthetic T_1_-weighted images at different TIs, 16 synthetic T_2_-weighted images at different TEs, and six shape prior probabilities of the different tissue classes (fat, muscle, prostate, bone marrow, bladder, and air).

Each of the 36 features is normalized [28] so they have zero mean and unit variance:

$$f_{norm}=\frac{f_{old}-\mu }{\sigma }$$ (4)

where f_old_ is the old feature value, f_norm_ is the normalized value, and μ and σ are the mean and standard deviation of the original feature range. The main advantage of normalization is to avoid features spanning across a large numeric range to dominate those with a smaller numeric range.

### Classification

The tissue classification is performed using the multi-class linear SVM implementation from the libLinear software package [19]. A One-vs-All method was used to solve the multi-class classification problem. It consists in developing for each class a binary classifier that separates that class from the rest of the data. A linear SVM kernel was chosen due to the lack of prior data suggesting the use of a non-linear SVM kernel (with our features), and due to the lower vulnerability for overfitting compared with non-linear kernels [29]. The method then combines the classifiers for multi-class inference [30].

### Evaluation

To evaluate the performance of the multi-class SVM classifier, a leave-one-out cross-validation was implemented. The overall database contains images from 22 subjects. We repeatedly trained our method on data from 21 subjects and tested on the remaining one. The reported cross validation accuracy is the percentage of data correctly classified pixel-by-pixel.

By its very nature, the dataset is highly imbalanced with far more pixels from the fat, muscle and bone marrow classes than from the prostate, bladder and air classes. To avoid biasing the classification toward the larger classes, the number of voxels from the fat, muscle and bone marrow classes were randomly down-sampled to match the number of voxels in the prostate class. Since the bladder and air ROIs are smaller than the prostate, we kept every pixel from those classes.

The mean accuracy was calculated for different combination of features to find the one with the higher prediction accuracy. Note that the classification process predicts a class label for each pixel, but accuracy is reported based on the ROIs manually outlined by the radiologist as shown in Fig 2.

## Results

The segmentation-classification accuracy for the different tissues with different feature combinations is shown in Table 1. For most of the SVM models, the prostate classification accuracy was excellent (>80%), except for some models using a single feature descriptor (i.e. T_1_ relaxation time (≈ 72%), T_2_ relaxation time (0%) or synthetic T_1_-weighted images (≈ 75%)). The classification accuracy of fat and muscle was also excellent (>90%), except for the model using only the shape prior as feature descriptor (< 80%). The classification accuracy of bone-marrow only achieved good results in SVM models including the shape prior feature (> 85%), otherwise the SVM classification failed (< 25%). The bladder classification accuracy was excellent (> 80%) for those SVM models including most of the features (i.e. synthetic T_1_- and T_2_-weighted and shape prior; and all the features). Please note that the reported accuracy of the bladder was computed on the slices in which it is visible. The classification of air within the body was not always satisfactory (<48%) in all the SVM models, mainly due to the small size of this region and the difficulty to model air signal (classified as noise). The use of synthetic T_1_- and T_2_-weighted features resulted in an overall better segmentation-classification accuracy than just using T_1_ and T_2_ relaxation times as features descriptors for the SVM classifier.

**Table 1:** Classification accuracy.

Combination of features	Classification accuracy (mean)					
	Prostate	Fat	Muscle	Bones	Bladder	Air
T1 Relaxation time	72.49	99.24	15.87	0.40	0.00	0.00
Synthetic T1-weighted	75.31	93.83	88.41	17.14	30.35	0.38
T2 Relaxation time	0.00	97.70	98.87	0.00	0.00	0.00
Synthetic T2-weighted	82.92	93.97	90.00	0.00	18.43	13.38
T1 and T2 Relaxation times	87.48	97.76	95.88	0.00	0.00	0.00
Synthetic T1- and T2-weighted	91.68	94.04	94.46	24.46	42.27	14.05
Shape prior	93.54	78.35	75.61	87.32	62.46	0.00
T1 Relaxation time and shape prior	93.73	96.37	93.35	89.59	64.87	11.50
Synthetic T1-weighted and shape prior	93.03	96.48	94.83	90.02	66.35	9.50
T2 Relaxation time and shape prior	92.41	95.03	95.00	89.35	62.69	9.12
Synthetic T2-weighted and shape prior	93.56	95.71	95.25	90.19	65.11	35.28
T1 and T2 Relaxation times + Shape prior	93.13	96.62	95.43	89.66	67.60	29.35
Synthetic T1- and T2-weighted and Shape prior	94.41	96.85	95.93	91.03	83.60	43.58
All features	94.23	96.90	95.89	91.05	82.10	47.55

Accuracy for SVM classification with leave-one-out validation for prostate, fat, muscle, bone marrow, bladder and air, including the list of the feature combination for each of the models. The overall classification accuracy is excellent (above 80%) for most regions when using at least a combination of 3 features. Results for the air class are less accurate, mainly because of the small size of that region.

**Fig 6:**
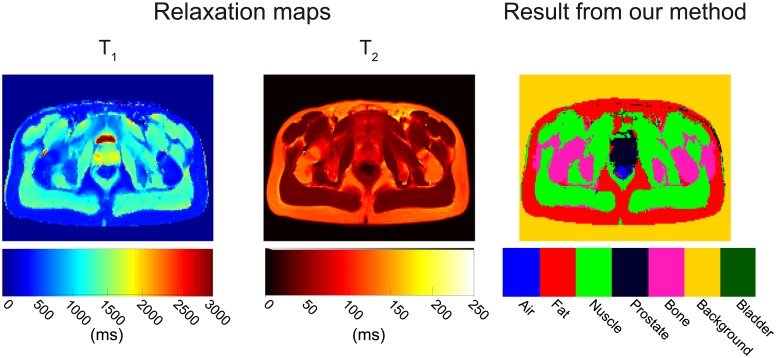
Relaxation maps and segmentation map of volunteer 1. Both T_1_ and T_2_ relaxations maps show homogeneous time values within the area of each tissue: prostate, muscle, fat, bladder and bone marrow. In the T_1_ and T_2_ maps, fat and bone marrow are not visually separable. The same happens in the T_2_ map for prostate and fat, muscle and bladder. However, the SVM classifier accuracy is rather excellent for prostate = 99.6%, fat = 92.5%, muscle = 99.3%, and bone marrow = 96.7%. Accuracy is lower for bladder (10.4%) and air (45%) mostly because of the small size of these regions.

Fig 6 presents an example of the computed T_1_ and T_2_ relaxation maps and the corresponding segmentation-classification results using all features. Visual inspection of the T_1_ map indicates a clear difference among the relaxation times of prostate (≈1700 ms), muscle (≈ 1500 ms), fat (≈ 380 ms) and bladder (≈ 2950 ms) so tissues from the various classes could easily be linearly separated. However, in the T_2_ map, the differences among the relaxation times of prostate (≈ 70 ms)—fat (≈ 90 ms)—bladder (≈ 60 ms) and fat—bone marrow (≈ 95 ms) are not visually perceptible. Nevertheless, the segmentation-classification accuracy for prostate, muscle, fat and bone marrow was excellent (≥ 90%). The bladder and air segmentation-classification accuracy was less accurate with around 10 and 45%, respectively.

**Fig 7:**
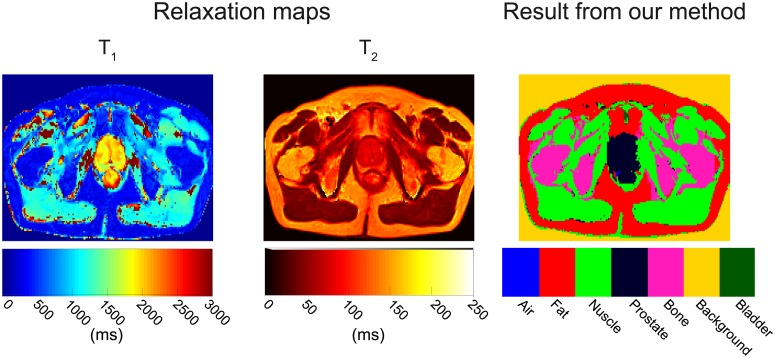
Relaxation maps and classification results of volunteer 3. Even though this volunteer is affected by noncancerous prostatic hyperplasia, the T_1_ map presents a rather homogeneous range of values in the prostate area (1600–2000 ms). The T_1_ map also presents a considerable amount of misleading values (≥2000 ms) in the muscle areas. On the T_2_ map, the muscle areas are well defined (<70ms) and clearly differentiable from the rest of the tissues (prostate, fat, and bone marrow) which seem to merge in a single region. In any case, the SVM accuracy is excellent: prostate = 89.1%, muscle = 98.8%, fat = 100%, and bone marrow = 89.7%. Bladder and air are not present. All features were used to perform the classification.

Another example of the T_1_ and T_2_ relaxation maps and segmentation-classification results is shown in Fig 7 for a volunteer affected with benign prostatic hyperplasia. The T_1_ relaxation map has considerably spurious relaxation times (≥2000 ms) in the muscle areas, and as expected fat and bone marrow are not easily differentiated. In the T_2_ relaxation map, only two areas are observable: 1) values below 70 ms (muscle) and 2) values above 71 ms (prostate, fat, bone marrow and feces). However, despite these difficulties the segmentation-classification accuracy for prostate (89.1%), muscle (98.8%), fat (100%) and bone marrow (89.7%) was high.

**Fig 8:**
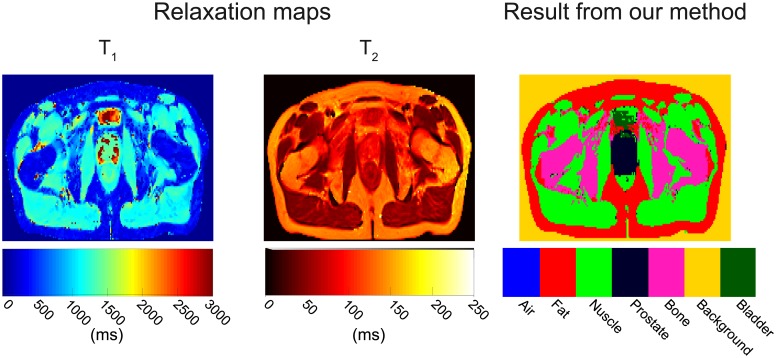
Relaxation maps and classification results of volunteer 20. The T_1_ relaxation map shows heterogeneous values for the bladder and prostate areas, and homogeneous non-distinctive areas for fat and bone marrow which complicate the classification process. The T_2_ map only shows two discernible areas: 1) fat-bone marrow-prostate-bladder (≥100ms) and 2) muscle (<100 ms) adding additional complexity to the classification process. Nevertheless, the SVM classifies accurately labeled the different areas: prostate = 89.1%, fat = 100%, muscle = 98.8%, bone marrow = 89.7%, bladder = 71.9% and air was not present. All features were used to perform the classification.

A particular example of the T_1_ and T_2_ relaxation maps and classification-segmentation results is shown in Fig 8. For this volunteer, the prostate does not present a homogeneous set of T_1_ relaxation times. Its values overlap those of muscle and bladder (1300 ms to 3000 ms). In the T_2_ relaxation map, the prostate values overlap those of fat and bladder (≥ 100 ms). However, the bladder is easily differentiated in the T_1_ relaxation map, but not in the T_2_ relaxation map (i.e. its values overlap with those of prostate and fat). Nevertheless, the good accuracy of our method for prostate (89.1%), bladder (71.9%) and fat (100%) shows that a linear classifier works well, even for challenging images.

## Discussion

In this paper, we presented a new methodology to classify tissues within the pelvic. The approach uses a multi-class linear SVM and MRI relaxation times features to segment prostate, fat, muscle, bone marrow, bladder and air. The overall accuracy of the method indicates that our approach is a viable option to automatically classify and segment these tissues and that the proposed features allow to linearly separate tissues. The lower accuracy for bladder and air compared with the other tissues is due to the lack of enough training and testing data for those two tissues (besides, they were not always visible in all imaged slices). Since the performances of SVM depend on the availability of training data, the lack of enough samples of bladder and air affects its performance. Also, since air and bladder cover a small area, an error of just a few millimeters drastically reduces accuracy, especially when T_1_- and T_2_-weighted images are not rigorously aligned. A possible solution for the lack of samples is data augmentation, i.e. bladder and air samples could be cloned with random distortions to increase the number of training samples. As for the misalignment, it can be due to rectal distension and rectal contraction between acquisitions. The implementation of a non-rigid registration technique could be a solution for this problem. The air classification accuracy also depends on the capacity of the SVM classifier to model the air signal. However, if the air signal is present, it is classified as noise (air does not give any signal when exposed to the radio frequency pulse of MR imaging). Therefore, the SVM classifier is trying to model a noisy signal to classify air. Since noise is formed randomly during each acquisition, its modeling is challenging. Thus, this could be another reason for the low performance of the air classification accuracy.

The accuracy of the SVM classifier also depends on the chosen feature descriptors. The feature descriptors are T_1_ relaxation time, synthetic T_1_-weighted images, T_2_ relaxation time, synthetic T_2_-weighted images and the tissue prior probabilistic maps. The accuracy of the results indicates that the chosen feature descriptors characterize well the variability of the tissue characteristics.

The T_1_ and T_2_ relaxation times are optimal feature descriptors because they represent physical properties that can be compared between cohorts. They are independent of scanner’s hardware and acquisition protocol, and they can be used to generate synthetic weighted images.

The synthetic T_1_- and T_2_-weighted images have been used as a "perfect" tissue descriptor because they are independent of hardware and artifacts affecting MR image reconstruction. They can generate a specific TI and TE to achieve reliable differentiation of all desired tissues. This is unlike combining several contrast weighting techniques which is usually insufficient to distinguish tissues [6]. When generated with the correct TIs or TEs sample points, synthetic weighted-images bring to light the subtle differences between tissues with close relaxation times, which help to increase classification accuracy. In fact, the inclusion of the synthetic weighted images as features increased the bladder classification accuracy to satisfactory levels (>80%), which was not possible using only the T_1_ and T_2_ relaxation times (<68%). Moreover, synthetic images can be generated at the same relaxation sample point for every subject. This forms a common framework which increases the intra- and inter-scanner reproducibility, which could certainly help to carry out multi-center studies. Multi-center studies are difficult because merging data across scanners is problematic (i.e. the way tissues’ properties are changed to signal intensities is scanner dependent rather than tissue dependent [3], and these signal intensities are arbitrary). However, the standardized nature of synthetic weighted images facilitates comparison across sites and time points.

The diagnostic value of synthetic T_1_- and T_2_-weighted images was not thoroughly evaluated although their quality when compared to conventional MR images has been shown sufficient [31,32]. Future research could further explore the true diagnostic value of synthetic images. Also, while this work focuses on relaxation time features, it could be easily extended to work with other tissues’ physical properties such as the apparent diffusion coefficient, which could be used to generate synthetic diffusion weighted images.

Tissue segmentation and classification with machine learning and relaxation times features has received increasing interest recently. The work that relates the most to our study is a decision tree model method of tissue segmentation and classification based on the T_1_ and T_2_ relaxation times and anatomical knowledge proposed in [24]. In their study, prostate, fat, muscle and bone marrow are segmented and classified. However, their study does not take advantage of the relaxation time capacity to generate synthetic T_1_- and T_2_-weighted images neither does it go further to localize other body regions (i.e. bladder and air). Moreover, their method uses a special procedure to localize bone marrow, while in our method bone marrow is localized directly by the SVM classifier.

The corner stone of the proposed method are the T_1_ and T_2_ relaxation times, for this reason an inversion recovery and spin echo sequences were used to compute them. These sequences are considered as the gold standard for MRI relaxometry. However, the trade-off is a long acquisition time required to acquire the T_1_- and T_2_-weighted images, which could be a difficulty in clinical practice. Hence, faster sequences could be adapted to compute the relaxation times in the pelvis (e.g. the Variable Flip Angle steady state spoiled gradient recalled echo (SPGR) imaging technique provides a series of high resolution T_1_-weighted images in a clinically feasible time [35]), and the method should work with these sequences thus it mostly depends on the relaxation times to perform the segmentation-classification, i.e. it is sequence independent.

Age related modifications in relaxation times are complex, but can be summarized as (i) a decrease in the degree of hydration of tissues with age and (ii) an increase in fat content in muscles [36,37]. A decrease in tissue hydration will lead to shorter T1 and especially T2 relaxation times in tissues, whereas an increase in tissue fat content will reduce T1, but increase T2. However, it could be infer that these variations are modest thus the SVM classification accuracy was excellent for muscle, fat, and bone marrow for the range of ages involved in the study (22–63 years old), since these tissues are the most affected by age related modifications in their relaxation times. Nevertheless, a bigger cohort with an ample range of ages is necessary to validate how well the SVM method can cape with these T1 and T2 age related variations.

Moreover, classification of prostate cancer tissue is not addressed in this study and an additional type of tissue (corresponding to prostate cancer) should be considered with a T1 slightly higher and a T2 considerably lower than the ones of normal prostate gland.

The main limitation of this study is the small number of volunteers. Twenty-two subjects are not enough to claim generalization, even though the fact that the SVM results came from a careful leave-one-out cross validation which reduces the bias of the reported accuracy values. Further validation on a much larger cohort that would include man of all ages, races and body masses is necessary.

## Conclusion

We presented a reliable multi-linear SVM method to segment and classify the structures present in prostate MR examination: prostate, fat, muscle, bone marrow, bladder and air using the intrinsic T_1_ and T_2_ relaxation times of tissues. The SVM results provide solid information about these structures and could be potentially useful to measure the prostate volume for radiotherapy applications or for multi-modality registration (e.g. PET/MRI) to generate attenuation maps, and to perform quantitative analyses.
